# Capturing Complex 3D Human Motions with Kernelized Low-Rank Representation from Monocular RGB Camera

**DOI:** 10.3390/s17092019

**Published:** 2017-09-03

**Authors:** Xuan Wang, Fei Wang, Yanan Chen

**Affiliations:** 1 The Institute of Artificial Intelligence and Robotics, Xi’an Jiaotong University, No.28 Xianning West Road, Xi’an 710048, China; xwang.cv@gmail.com (X.W.); chenyanan@stu.xjtu.edu.cn (Y.C.); 2 The School of Software Engineering, Xi’an Jiaotong University, No.28 Xianning West Road, Xi’an 710048, China; 3 National Engineering Laboratory for Visual Information Processing and Application, Xi’an Jiaotong University, No.28 Xianning West Road, Xi’an 710048, China; 4 Shaanxi Digital Technology and Intelligent System Key Laboratory, Xi’an Jiaotong University, No.28 Xianning West Road, Xi’an 710048, China

**Keywords:** 3D human pose estimation, monocular reconstruction, non-rigid structure from motion, kernel low-rank representation

## Abstract

Recovering 3D structures from the monocular image sequence is an inherently ambiguous problem that has attracted considerable attention from several research communities. To resolve the ambiguities, a variety of additional priors, such as low-rank shape basis, have been proposed. In this paper, we make two contributions. First, we introduce an assumption that 3D structures lie on the union of nonlinear subspaces. Based on this assumption, we propose a Non-Rigid Structure from Motion (NRSfM) method with kernelized low-rank representation. To be specific, we utilize the soft-inextensibility constraint to accurately recover 3D human motions. Second, we extend this NRSfM method to the marker-less 3D human pose estimation problem by combining with Convolutional Neural Network (CNN) based 2D human joint detectors. To evaluate the performance of our methods, we apply our marker-based method on several sequences from Utrecht Multi-Person Motion (UMPM) benchmark and CMU MoCap datasets, and then apply the marker-less method on the Human3.6M datasets. The experiments demonstrate that the kernelized low-rank representation is more suitable for modeling the complex deformation and the method consequently yields more accurate reconstructions. Benefiting from the CNN-based detector, the marker-less approach can be applied to more real-life applications.

## 1. Introduction

Most of the video or image data used nowadays are captured by a single RGB sensor, such as cell phone cameras, which is one of the most widely used sensors. Recovering the 3D structure from such data is an active research field in computer vision and computer graphics communities. In this work, we focus on tackling the problem of reconstructing the complex 3D motions, especially for the 3D human poses, from the monocular image sequence. This is an inherently ill-posed problem since the same projection can be captured from different 3D structures. To resolve the ambiguities, several approaches relying on additional priors have been proposed. To be specific, lots of methods have been presented to solve the 3D human pose estimation problem, which leverage more priors from human bodies.

The seminal factorization [1] of Non-Rigid Structure from Motion (NRSfM) techniques was proposed to tackle the non-rigid problem via extending its rigid version [2]. In Xiao et al. [3], the shape basis constraints is presented to resolve the inherent ambiguities and derived the closed-form solution. Akhter et al. [4,5] showed the dual approach which modeled 3D trajectories under basis constraints. In addition, the trajectory-space method was proposed in Gotardo and Martinez [6]. In Akhter et al. [7], it proved that, even though there is an ambiguity in shape bases or trajectory bases, non-rigid shapes can still be recovered uniquely. Based on this, a prior-free method [8] was introduced to estimate the 3D non-rigid structures and camera rotations by only exploiting the low-rank shape assumption. In Wang et al. [9], they use the low-rank assumption in a similar way, but an Accelerated Proximal Gradient (APG) algorithm solver is employed to solve the resulting problem. Furthermore, the method in Gotardo and Martinez [10] combined the shape basis model and trajectory basis model, and revealed trajectories of the shape basis coefficients. The Procrustean Normal Distribution (PND) model was proposed in Lee et al. [11], where 3D shapes are aligned and fit into a normal distribution. Simon et al. [12] exploited the Kronecker pattern in shape-trajectory (spatial-temporal) priors. Then, Zhu and Lucey [13] combined the convolutional sparse coding technique with NRSfM by using point trajectory. Nevertheless, it requires learning an over-complete basis of trajectories. Most recently, a novel part-based method has been proposed in Lee et al. [14]. For most existing approaches mentioned above, the performance highly depends on the complexity of the 3D non-rigid motions. Generally, the correspondences between different frames are assumed to be given, by e.g., infrared markers. As a consequence, they usually fail to handle the reconstruction problem in uncontrolled scenarios with complex non-rigid motions.

The human body is an important subject in nonrigid reconstruction since reconstruction of 3D human structures has broad applications, such as athletic training, computer animation and gait analysis. Using multi-camera systems [15,16] can easily resolve the ambiguities in non-rigid reconstruction problems, and the requirement for dedicated equipment prevents the methods from applying in more practical scenarios. Using a depth camera can also avoid the ambiguities in monocular 3D non-rigid reconstruction problems [17]. Since 3D information can be directly obtained by depth-cameras, the problem of recovering 3D human poses can be formulated as problem of 3D tracking [18]. However, massive existing videos are captured by the single RGB camera. Therefore, estimating 3D human structures from monocular image sequence is still an essential task. In Rádlová et al. [19], a model-based approached was presented. According to Sigal [20], estimating the accurate pose on single frame is an ill-posed problem, and exploiting all available information across the sequence can promote performance [21,22]. Latent Variable Models (LVMs) are often used in the literature [23,24]. Tian et al. [25] proposed a discriminative approach that leverages LVMs, and successfully addressed the over-fitting and poor generalization problem. In Zhu et al. [26], the human body is classified into several parts; a pre-defined human model is fitted to the input images; and the dense reconstruction is yielded. Ek et al. [27] presented a method relying on the Gaussian process latent variable models (GPLVM), in which a parametric mapping from pose to latent space is learned to enforce a one-to-one correspondence. In the work of Tekin et al. [28], they employed two convolutional neural networks to align the bounding boxes of the human in consecutive frames, then created the data volume and reconstructed the 3D structure from the volume with Kernel Ridge Regression (KRR) and Kernel Dependency Estimation. With the advent of deep learning, it is obvious that using CNN to obtain the human joint detection or to estimate the 3D structures directly attracts more and more attention [29]. For more details, a comprehensive survey can be found in Gong et al. [30].

In this paper, we present an NRSfM method to tackle the problem of recovering complex 3D non-rigid motions from a monocular sequence. The term ’complex’ here means that the entire motions are composed of several different ’primitive’ or ’simple’ actions. The proposed method empirically shows that the assumption, i.e., 3D shapes lie on the union of nonlinear shape subspaces, is better than the linear version [31] in modeling complex 3D non-rigid motions. We formulate this assumption as kenerlized low-rank representation and model the motion recovery as solving an optimization problem. The experiments demonstrate that our method has better performance than the state-of-the-art methods, especially for tackling the complex motions. Moreover, using the outputs, called joint heightmaps in this paper, of a CNN-based human joint detector [32] can remove the dependency on markers. As a result, our method can be applied to more practical scenarios. We can conveniently switch between the joint heightmap based data term and the traditional re-projection term. This yields two versions of our method—marker-based and marker-less versions—and ensures the method can be applied more broadly.

## 2. Motivations

Before we introduce the proposed method, let us give a brief review of two lines of research that are most relevant to our method. One is the NRSfM based on low-rank shape constraints, and the other is low-rank representation.

### 2.1. NRSfM with Low-Rank Priors

In Dai et al. [8], an NRSfM method only relying on low-rank priors was proposed. Given the tracked feature points, e.g., human joints in images, the reconstruction is modeled as the following optimization problem:

(1) $$\begin{array}{cc} \min_{X} & \frac{1}{2}{∥W-RX∥}_{F}^{2}+\mu {∥X^{\#}∥}_{*}\\ s.t. & X^{\#}=g\left(X\right), \end{array}$$

where $X\in {ℜ}^{3N_{P}\times N_{F}}$ is the 3D structure matrix to be estimated, which stacks 3D coordinates of all the $N_{P}$ feature points at all the $N_{F}$ frames. The first term in Equation (1) is a re-projection term that encourages projections of the estimated 3D structures to be close to the observations in W. In this term, $W\in {ℜ}^{2N_{P}\times N_{F}}$ and $R\in {ℜ}^{2N_{F}\times 3N_{F}}$ are observation matrix and rotation matrix, which encode the camera rotations and 2D positions of tracked feature points (human joints for human pose estimation), respectively. The second term encourages the 3D structures at all the frames to be low-rank. In other words, all the 3D structures can be represented by linear combinations of a few 3D structure bases, the number of which is much smaller than the number of the frames. Note that the shape basis is not obtained explicitly here. By contrast, the nuclear norm used in the first term ensures that estimated 3D structures should be as low-rank as possible. The relative weight μ is to control how strong this prior influences the resulting reconstruction. Therefore, this makes the original monocular non-rigid reconstruction problem well-constrained. However, when motions are complex, 3D structures are quite different from each other, and the low-rank assumption is broken since 3D structures don’t lie on a single low-rank linear space. In this case, either a high-rank shape space or multiple subspaces are needed to represent the 3D structures.

### 2.2. Low-Rank Representation

Low-rank representation (LRR) [33] was proposed to seek the desired low-rank affinity matrix Z via solving the optimization problem in Equation (2):

(2) $$\begin{array}{cc} \min_{Z,E} & {∥Z∥}_{*}+\lambda {∥E∥}_{2,1}\\ s.t. & X=XZ+E, \end{array}$$

where X is data matrix and E is error matrix. In particular, LRR uses the self-expressiveness property of data, i.e., each data point in a union of subspaces can be efficiently represented as a linear combination of other points. When pursuing the low-rank affinity matrix, the data points are assigned to different subspaces implicitly. This property ensures LRR can be an effective regularization to handle the complex motions in NRSfM problem [31]. To this end, the formulation is as follows:

(3) $$\begin{array}{cc} \min_{X,Z,E} & {∥Z∥}_{*}+\lambda_{1}{∥X∥}_{*}+\lambda_{2}{∥E∥}_{l}\\ s.t. & X=XZ,\;W=RX^{\#}+E. \end{array}$$

Nevertheless, the deformation of non-rigid object, e.g., human body, is often nonlinear. To better model the nonlinear deformation, the traditional approach is mapping the data point to a higher-dimension space in which the linear low-rank structure can be obtained. We empirically find that the kernerlized low-rank representation (KLRR) method [34] has better performance on human motion clustering than original LRR. To this end, we introduce the KLRR as a regularization to the NRSfM problem.

## 3. Formulations

In order to recover the highly complex 3D motions (also named structures, shapes or deformations) from a monocular image sequence, we map the 3D structures to a space with higher dimension, as shown in Equation (4)

(4) $$\begin{array}{cc} \min_{X,Z,E} & {∥Z∥}_{*}+\lambda_{1}∥X∥+\lambda_{2}{∥E∥}_{1}+\lambda_{3}{∥\Phi \left(X\right)\left(I-Z\right)∥}_{F}^{2}\\ s.t. & W=RX^{\#}+E, \end{array}$$

(5) $$\begin{array}{cc} X=\left[\begin{array}{cccc} x_{1,1} & x_{1,2} & \cdots & x_{1,N_{F}}\\ x_{2,1} & x_{2,2} & \cdots & x_{2,N_{F}}\\ \vdots & \vdots & \ddots & \vdots \\ x_{N_{P},1} & x_{N_{P},2} & \cdots & x_{N_{P},N_{F}} \end{array}\right], & X^{\#}=\left[\begin{array}{cccc} x_{1,1} & x_{2,1} & \cdots & x_{N_{P},1}\\ x_{1,2} & x_{2,2} & \cdots & x_{N_{P},2}\\ \vdots & \vdots & \ddots & \vdots \\ x_{1,N_{F}} & x_{2,N_{F}} & \cdots & x_{N_{P},N_{F}} \end{array}\right]. \end{array}$$

We follow the definition of notation above. Assuming the object is human body whose motions are captured by an orthographic camera, the aim of the proposed method is to recover the 3D position of its joints from the given 2D location of joints only. $X\in {ℜ}^{3N_{P}\times N_{F}}$ is the 3D motion matrix; here, the 3D coordinates are defined in the camera coordinate system. In both matrices X and $X^{\#}$, vector $x_{p,f}$ stacks the 3D coordinates of $p_{th}$ joint at frame *f*, but they have different arrangement of these vectors. R is the ${ℜ}^{2N_{F}\times 3N_{F}}$ block-diagonal matrix of $N_{F}$ orthographical camera matrices. The given 2D tracks of all the joint points are in the observation matrix $W\in {ℜ}^{2N_{F}\times N_{P}}$, and E is an error matrix with the same dimensions of W. The Φ(X) is the defined mapping, such that the mapped X resides in multiple linear subspaces. In addition, the matrix $Z\in {ℜ}^{N_{F}\times N_{F}}$ is the affinity matrix, i.e., the self-expressive coefficient matrix. Note that the noise in this problem usually occurs at the tracking or joint detection process. In this work, the part of tracking or detection is assumed to be given by other existing methods. Certainly, such tracking or detection approaches could not yield the perfectly accurate results. Thus, when dealing with the re-projection constraint, we use the matrix E to model the possible re-projection error. Furthermore, we penalise the more robust $L_{1}$ norm rather $L_{2}$ norm of this matrix. It ensures that the method is relatively robust to the outliers of observation.

The low-rank shape priors is preserved in our framework. In Equation (4), $\lambda_{1}{∥X∥}_{*}$ is to exploit the low-rank nature of non-rigid objects, and the constraint $W=RX^{\#}+E$ is used to penalize the re-projection error. Note that nuclear norm, a convex approximation of matrix rank, of X, rather than $X^{\#}$, is minimized here, as described in Dai et al. [8]. This is because the rank of X is bound by min$(N_{F},3N_{P}),$ whereas the rank of $X^{\#}$ is bound by min$\left(3N_{F},N_{P}\right)$. Minimizing the rank of X is preferable as it attempts to directly learn redundancies among frames.

The information revealing the low-rank subspaces structure exists in the affinity matrix Z. By applying the spectral clustering to Z, the explicit structure can be obtained. More details about this process can be found in [33,34]. Nevertheless, our method doesn’t need the explicit subspace structure. It only needs to ensure that the 3D structures from a lower rank subspaces are preferred by minimizing the nuclear norm of Z. If the low-rank subspaces assumption holds, this term will be a proper regularization for NRSfM problem with complex 3D motions. Unfortunately, LRR may not yield satisfied results when the 3D structures are actually from the nonlinear subspaces, since it is originally designed to handle the linear case.

Due to this drawback of LRR, we exploit the kernel-induced mapping X→Φ(X). We denote the column in X as ${\left\{x_{i}\right\}}_{i=1}^{N_{F}}$, where $x_{i}\in {ℜ}^{3N_{P}}$, define the kernel matrix $K\in {ℜ}^{N_{F}\times N_{F}}$, and then the elements of *K* are calculated as follows:

(6) $$\begin{array}{c} K_{ij}=k\left(x_{i},x_{j}\right),\;\;\forall i,j=1,\cdots ,N_{F}, \end{array}$$

where $k\left(x_{i},x_{j}\right)=\phi {\left(x_{i}\right)}^{T}\phi \left(x_{j}\right)$ is the kernel function. Defining $\Phi \left(X\right)=[\phi \left(x_{1}\right),\cdots ,\phi \left(x_{N_{F}}\right)]$ , we have $K\left(X\right)=\Phi {\left(X\right)}^{T}\Phi \left(X\right)$. In the remainder of this paper, we use K to represent K(X) for short. We can rewrite Equation (4) as follows:

(7) $$\begin{array}{cc} \min_{X,Z,E} & {∥Z∥}_{*}+\lambda_{1}∥X∥+\lambda_{2}{∥E∥}_{1}+\lambda_{3}trace\left({\left(I-Z\right)}^{T}K\left(I-Z\right)\right)\\ s.t. & W=RX^{\#}+E. \end{array}$$

Equation (7) above is designed for the general purpose. When considering the special case, e.g., recovering the 3D motions of human bodies, we can leverage additional particular priors. The exploited prior is that the length between the connected joints is invariable. Given the set of all the pairs of connected joints E, the inextensibility constraints can be written as:

(8) $$\begin{array}{c} R\left(X,l\right)=\sum_{f=1}^{N_{F}}\sum_{(p,q)\in E}(∥x_{f,p}-x_{f,q}{{∥}_{2}-l_{p,q})}^{2}, \end{array}$$

where $x_{f,p}$ stacks the 3D coordinates of point *p* at frame *f*. The $l=\{l_{p,q}\}$ are unknown, in which $l_{p,q}$ encodes the length between the pairs of connected joints *p* and *q*, and thus act as the auxiliary variables to be determined by our algorithm. Adding Equation (8) into Equation (7), we have the final objective function as follows:

(9) $$\begin{array}{cc} \min_{X,Z,E,l} & {∥Z∥}_{*}+\lambda_{1}∥X∥+\lambda_{2}{∥E∥}_{1}+\lambda_{3}trace\left({\left(I-Z\right)}^{T}K\left(I-Z\right)\right)+\lambda_{4}R\left(X,l\right)\\ s.t. & W=RX^{\#}+E. \end{array}$$

In this formulation, the observation matrix *W* is often obtained by setting markers on the subject. Nevertheless, in some daily-life scenarios, e.g., recovering 3D human motions using a camera on the mobile phone, markers are usually unavailable. To this end, we provide an optional version of our method in which an CNN-based human joint detector [32] is employed. By a small modification, Equation (9) can be conveniently converted to a marker-less version as follows:

(10) $$\begin{array}{cc} \min_{X,Z,E,l} & {∥Z∥}_{*}+\lambda_{1}∥X∥+\lambda_{2}{∥E∥}_{1}+\lambda_{3}trace\left({\left(I-Z\right)}^{T}K\left(I-Z\right)\right)+\lambda_{4}R\left(X,l\right)+\lambda_{5}H\left(\hat{W}\right)\\ s.t. & W=RX^{\#}+E,W=\hat{W}, \end{array}$$

where the function $H(\hat{W})$ is yielded by the CNN-based detector. For arbitrary joint point *p* and frame *f*, the CNN-based detector provides a heightmap $h_{f,p}$ with the same size of input image. The pixel in the heightmap $h_{f,p}$ takes a non-negative value, which indicates how possible it is the projection of the joint *p* at frame *f*. Therefore, the function $H(\hat{W})$ is calculated as follows:

(11) $$H\left(\hat{W}\right)=-\sum_{f=1}^{N_{F}}\sum_{p=1}^{N_{P}}h_{f,p}\left({\hat{w}}_{f,p}\right),$$

where $\hat{W}=\left\{{\hat{w}}_{f,p}\right\}$ is the auxiliary variable introduced for the convenience of optimization. The ${\hat{w}}_{f,p}$ stacks the 2D image coordinate of the projection of 3D point $x_{f,p}$ .

## 4. Optimization

In the previous section, capturing 3D human motions from monocular images is formulated as solving the optimization problem (9). We use ALM to solve the yielding optimization problem. By introducing auxiliary variables C and $\hat{X}$, the complete Lagrangian formulation is:

(12) $$\begin{array}{cc} \min_{X,\hat{X},Z,E,C,l}\max_{\Gamma_{1},\Gamma_{2},\Gamma_{3}}L= & {∥Z∥}_{*}+\lambda_{1}∥\hat{X}{∥}_{*}+\lambda_{2}{∥E∥}_{1}+\lambda_{3}trace\left(C^{T}KC\right)+\lambda_{4}R\left(X,l\right)\\ & +<\Gamma_{1},W-RX^{\#}-E>+\frac{\mu }{2}{∥W-RX^{\#}-E∥}_{F}^{2}\\ & +<\Gamma_{2},C-I+Z>+\frac{\mu }{2}{∥C-I+Z∥}_{F}^{2}\\ & +<\Gamma_{3},X-\hat{X}>+\frac{\mu }{2}{∥X-\hat{X}∥}_{F}^{2}, \end{array}$$

where $\Gamma_{1}$ and $\Gamma_{2}$ are the Lagrangian multiplier matrices. In iterations of Algorithm 1, the variables {X,Z,E,C,l} are alternatively solved. When solving each subproblem, the other fixed variables are regarded as constants; hence, we ignore the iteration index for these variables. Then, at the end of each iteration, the penalty factor μ and Lagrangian multiplier matrices $\Gamma_{1}$, $\Gamma_{2}$ and $\Gamma_{3}$ are updated.

**Algorithm 1:** Solving problem (12) by ALM. **Input**: $W,R,\lambda_{1},\lambda_{2},\lambda_{3},\lambda_{4},\mu_{max},\rho$ **Output**: X,Z,E,C,l initialization $X^{0},Z^{0},E^{0},C^{0},l^{0},\Gamma_{1}=0,\Gamma_{2}=0,\mu_{0}=0,k=0$; **while**
*not converged*
**do** 
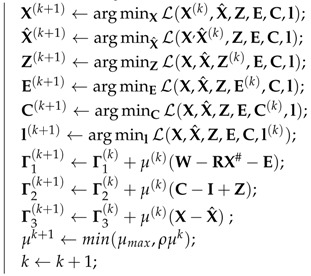
 **end**

### 4.1. The Solution of $\hat{X}$ and Z

By a simple derivation, the subproblems of solving Z and $\hat{X}$ can be written as the following optimization problems:

(13) $$\begin{array}{c} \min_{Z}{∥Z∥}_{*}+\frac{\mu }{2}{∥Z-\left(I-C-\frac{\Gamma_{2}}{\mu }\right)∥}_{F}^{2},\\ \min_{\hat{X}}\lambda_{1}∥\hat{X}{∥}_{*}+\frac{\mu }{2}{∥\hat{X}-\left(X+\frac{\Gamma_{3}}{\mu }\right)∥}_{F}^{2}, \end{array}$$

whose formulation is the same as that of the standard problem in Cai et al. [35]. For this reason, there are closed-form solutions for Z and $\hat{X}$. In the iteration *k*, these variables are updated by

(14) $$\begin{array}{c} Z^{\left(k+1\right)}=D_{1/\mu }\left(I-C^{\left(k\right)}-\frac{\Gamma_{3}^{\left(k\right)}}{\mu }\right),\\ {\hat{X}}^{\left(k+1\right)}=D_{\lambda_{1}/\mu }\left(X^{\left(k+1\right)}+\frac{\Gamma_{3}}{\mu }\right), \end{array}$$

where $D_{\tau }\left(\cdot \right)$ is the Singular Value Decomposition operator. The readers can refer to Cai et al. [35] for mode details.

### 4.2. The Solution of X

The subproblem of solving X has no closed-form solution due to the existence of non-convex terms. It is obvious that the subproblem can be converted to a nonlinear least square problem by the similar derivation, which is used to get Equation (13). Exploiting the gradient-based iterative optimization algorithm [36], we get the estimation of X at iteration *k*:

(15) $$\begin{array}{cc} X^{\left(k+1\right)}=\underset{X}{\mathrm{argmin}} & \lambda_{3}trace\left(C^{{\left(k\right)}^{T}}KC^{\left(k\right)}\right)+\lambda_{4}R\left(X,I^{k}\right)\\ & +\frac{\mu }{2}(∥X-{\hat{X}}^{\left(k\right)}+\frac{\Gamma_{3}^{\left(k\right)}}{\mu }{∥}_{F}^{2}+∥W-RX^{\#}-E^{\left(k\right)}+\frac{\Gamma_{1}^{\left(k\right)}}{\mu }{∥}_{F}^{2}). \end{array}$$

### 4.3. The Solution of E

Fixing X, $\hat{X}$, Z, C and l, the cost function is reduced to

(16) $$\begin{array}{c} \min_{E}\lambda_{2}{∥E∥}_{1}+\frac{\mu }{2}{∥W-RX^{\#}-E+\frac{\Gamma_{1}}{\mu }∥}_{F}^{2}. \end{array}$$

The resulting $l_{1}$ minimization problem has a closed-form solution:

(17) $$\begin{array}{c} E^{k+1}=S_{\lambda_{2}/\mu }\left(W-RX^{{\#}^{\left(k+1\right)}}+\frac{\Gamma_{1}^{\left(k\right)}}{\mu }\right), \end{array}$$

where $S_{\tau }\left(\cdot \right)$ is the element-wise shrinkage thresholding operator [37].

### 4.4. The Solution of C and l

Since K is semi-definite positive matrix, both subproblems for solving C and l are convex:

(18) $$\begin{array}{c} \min_{C}\lambda_{3}trace\left(C^{T}KC\right)+\frac{\mu }{2}{∥C-I+Z+\frac{\Gamma_{2}}{\mu }∥}_{F}^{2},\\ \min_{l}\lambda_{4}R\left(X,l\right). \end{array}$$

The subproblems have closed-form solutions. Setting the derivatives to zero first, we then solve the yielded linear least square problems to obtain the solutions:

(19) $$\begin{array}{c} C^{\left(k+1\right)}={\left(2\lambda_{3}K^{\left(k+1\right)}+\mu I_{N_{F}}\right)}^{-1}\left(\mu \left(I-Z^{\left(k+1\right)}\right)-\Gamma_{2}^{\left(k\right)}\right),\\ l_{p,q}^{\left(k+1\right)}=\frac{1}{N_{F}}\sum_{f=1}^{N_{F}}{∥x_{f,p}^{\left(k+1\right)}-x_{f,q}^{\left(k+1\right)}∥}_{2}. \end{array}$$

**Figure 1:**
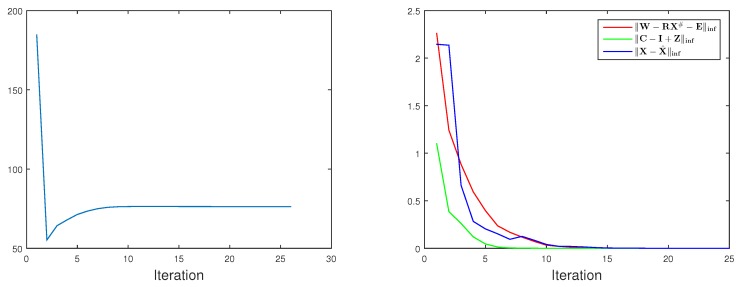
Convergence curve. We test our TUNS method on ’56-02’ sequence in CMU MoCap datasets is used here. We plot the residual of the objective function (**left**) and the residuals of three constraints (**right**).

For the existence of non-convex term and multiple blocks in our proposed objective function, the convergence of ALM is not guaranteed. Nonetheless, the Algorithm 1 successfully converged in our experiment as shown in Figure 1. For all the experiments, we leverage the resulting X from PTA [5] as the initialisation.

## 5. Experiments

We make the quantitative evaluation on two benchmarks: (i) synthetic camera 2D projections generated from the 3D CMU Motion Capture (MoCap) dataset, (ii) real-world 2D projection from the 2D point-tracks of video in Utrecht Multi-Person Motion (UMPM) [38] dataset. Furthermore, the performance of our marker-less algorithm is illustrated on the Human3.6M. We compare our approach, which is denoted as TUNS (temporal union of nonlinear subspaces) in the remainder of this section, against six NRSfM baselines: point tracking algorithm PTA [5], the trajectory-sapce method CSF [6], the block matrix method BMM [8], the temporal union of subspaces TUS [31], the accelerated proximal gradient optimization APG [9] and the consensus NRSfM of CNR [14]. For PTA [5], CSF [6], BMM [8], CNR [14], we use authors’ implementation in experiments. For PTA [5] and CSF [6], we manually set the rank of the subspace to the value yielding the best results. For TUS [31] and APG [9], since there are not publicly available implementations, our re-implementations are adopted in comparison. We test such re-implementations and get similar results to what the authors reported in [9,31].

### 5.1. Subspaces Analysis

**Figure 2:**
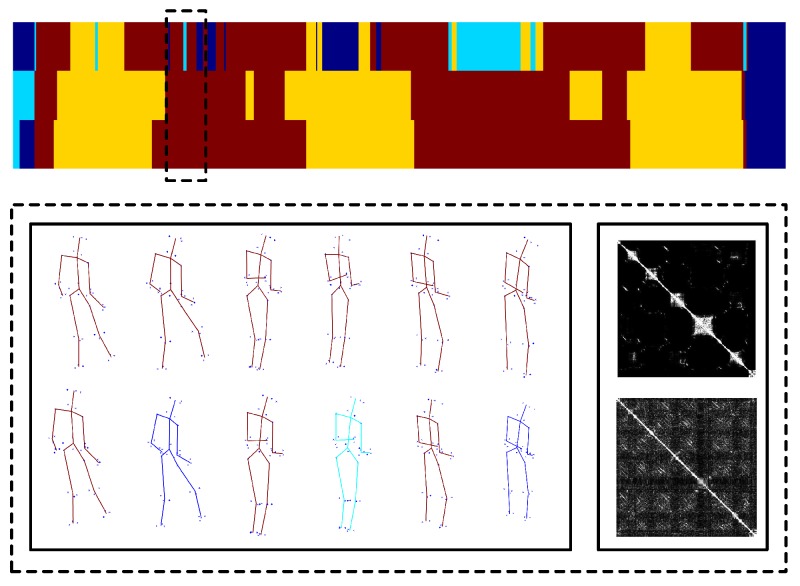
Subspace analysis on 3D human motions. On the top of this figure, three colour bars represent the subspace clustering results. In each bar, the same colour means same cluster. From the top down, the clustering results are obtained from the affinity matrices yielded by LRR [33], KLRR citeXiaoTNNLS15 and our method, respectively. For the first two rows, the LRR and KLRR are applied to the 3D motions ’p1_grab_3’ from the UMPM benchmark. For the third row, the affinity matrix produced from the reconstruction process is exploited. On the bottom, two rows of 3D motions and the clustering in a short and consecutive interval are visualized in a dash-line box. The first row is produced by KLRR and the second one is from LRR. The corresponding affinity matrices are shown on the right side.

We empirically demonstrate why the nonlinear low-rank representation could further help the reconstruction. To illustrate the effectiveness of utilising LRR and KLRR to recover the low-rank structures of 3D human motions, we select the motion sequence ’p1_grab_3’ from the UMPM benchmark. We apply both LRR and KLRR to the 3D motions to get the affinity matrices. Meanwhile, our reconstruction method also produces an affinity matrix. The difference is that LRR and KLRR estimate the affinity matrices on the 3D data directly; however, our method jointly estimates the 3D motions and affinity matrix from the 2D projection. Applying spectral clustering algorithm to these affinities, the motion clustering results are yielded. Obviously in Figure 2, the colour in the first row of the colour bars changes more frequently. This phenomenon is not preferable, since the image sequence is temporally consecutive. In other words, the LRR approach divides quite similar motions into different categories. Nevertheless, the clustering result based on KLRR, which has high temporal consistency, is quite close to the result of our method. As shown on bottom right of Figure 2, the affinity matrix from KLRR has higher clarity of the low-rank structure than the one from LRR. This experiment demonstrates that the nonlinear low-rank representation is more suitable to model the complex 3D motions than its linear counterpart.

### 5.2. Quantitative Evaluation

We quantitatively compare our TUNS approach with six baselines on 20 sequences of subjects 56 and 86, which contain several primitive actions, in CMU motion capture datasets. The synthetic 2D projections are generated by a randomly rotating orthographic camera, which rotates with the *y*-axis of camera and is always pointing at the centre of the body. Our TUNS approach and baseline methods are employed to reconstruct the 3D motions from the synthetic 2D projections. Since the monocular reconstruction has a global scale ambiguity, a type of mean relative reconstruction error $e_{mean}$, which is utilised in [8], is employed in this paper. This error is computed as follows:

(20) $$\begin{array}{c} e_{mean}=\frac{1}{\sigma N_{F}N_{P}}\sum_{f=1}^{N_{F}}\sum_{p=1}^{N_{P}}e_{fp},\\ \sigma =\frac{1}{3F}\sum_{f=1}^{F}\left(\sigma_{fx}+\sigma_{fy}+\sigma_{fz}\right), \end{array}$$

where $\sigma_{fx}$, $\sigma_{fy}$ and $\sigma_{fz}$ are the standard deviations in *x*, *y*, and *z* coordinates of the original shape at frame *f*. For each joint *p*, $e_{fp}$ is the distance between its reconstructed 3D position and its ground truth 3D position at frame *f*. Meanwhile, we report the median error which is computed as follows:

(21) $$e_{med}=median\left(\left\{e_{fp}|f=1\cdots N_{F},p=1\cdots N_{P}\right\}\right).$$

As reported in Table 1, our TUNS approach yields more accurate reconstructions than the baselines. PTA [5] can give a close-form solution that can be easily used as initialization for all the nonlinear optimization based algorithms. For CSF [6], it is not suitable for recovering the complex motion, since it has high space complexity with respect to the rank *K*. BMM [8] utilises no prior but the low-rank subspace. In such a method, the motions are assumed to lie in a single subspace. Consequently, it is less effective when recovering the complex motion. APG [9] inherently uses the single subspace, whereas it performs better than BMM [8] since a more effective rank-minimisation technique is employed. The part-based method CNR [14], which is more adept at handling complex shape configurations, yields better reconstruction of subject 86 than the subject 56, since sequences of subject 86 have more points than subject 56. Nonetheless, for the complex motions, using temporal union of subspaces (TUS and TUNS) can yield better reconstruction than the other methods. Benefiting from the kernel technique, our TUNS yields even more accurate reconstructions than TUS.

**Figure 3:**
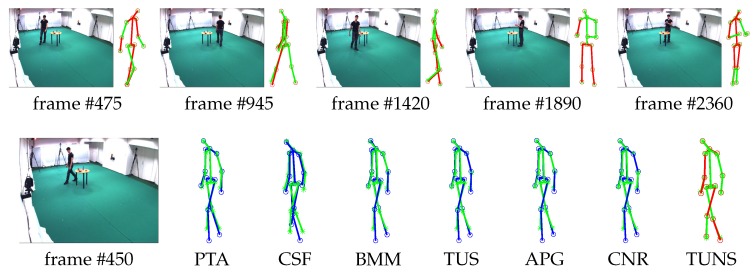
Evaluation on ’p1_grab_3’ sequence in UMPM. For five selected frames, our reconstruction (red skeleton), ground truth (green skeleton) and the corresponding image are shown on top. On the bottom, the reconstructions, yielded by all the baselines (blue skeleton) and our TUNS (red skeleton) of one specific frame, are shown with overlapping ground truth (green skeleton).

**Figure 4:**
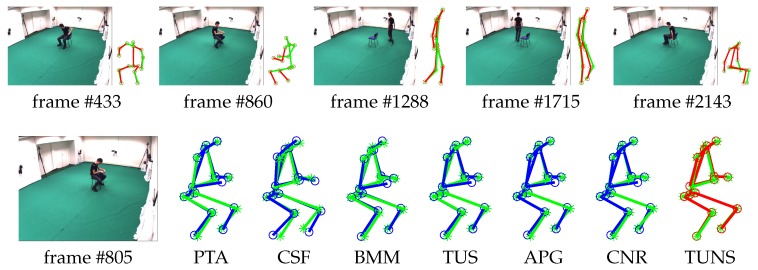
Evaluation on ’p1_chair_2’ sequence in UMPM. For five selected frames, our reconstruction (red skeleton), ground truth (green skeleton) and the corresponding image are shown on top. On the bottom, the reconstructions, yielded by all the baselines (blue skeleton) and our TUNS (red skeleton) of one specific frame, are shown with overlapping ground truth (green skeleton).

**Figure 5:**
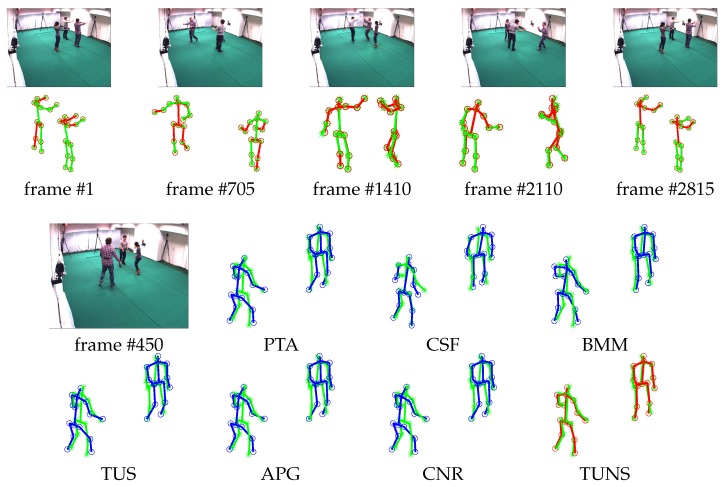
Evaluation on ’p3_ball_12’ sequence in UMPM. For five selected frames, our reconstruction (red skeleton), ground truth (green skeleton) and the corresponding image are shown on top. On the bottom, the reconstructions, yielded by all the baselines (blue skeleton) and our TUNS (red skeleton) of one specific frame, are shown with overlapping ground truth (green skeleton).

Meanwhile, we also evaluate the performance of our approach by reconstructing 3D motion from real-world 2D projection stemming from 2D point-tracks of videos in UMPM. Our TUNS and baselines are tested on the three sequences ’p1_grab_3’, ’p3_ball_12’ and ’p1_chair_2’. In Table 2, it shows that our method produces more accurate reconstructions. It demonstrates that TUNS is suitable for tackling the long-term sequence with complex motions. The reconstructions of some selected frames from UMPM datasets are shown in Figure 3, Figure 4 and Figure 5. Please note that Figure 5 illustrates that our TUNS approach is even effective in multi-object cases.

### 5.3. Qualitative Evaluation of Marker-Less Method

**Figure 6:**
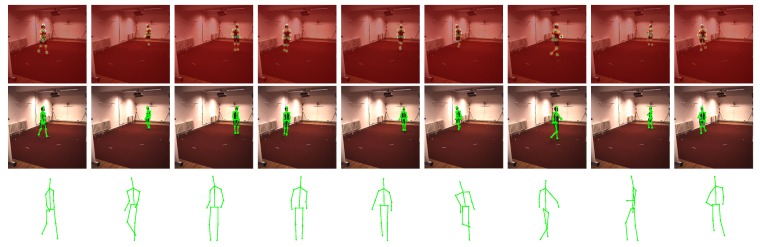
Evaluation on ’p3_ball_12’ sequence in UMPM. For five selected frames, our reconstruction (red skeleton), ground truth (green skeleton) and the corresponding image are shown on top. On the bottom, the reconstructions, yielded by all the baselines (blue skeleton) and our TUNS (red skeleton) of one specific frame, are shown with overlapping ground truth (green skeleton).

**Figure 7:**
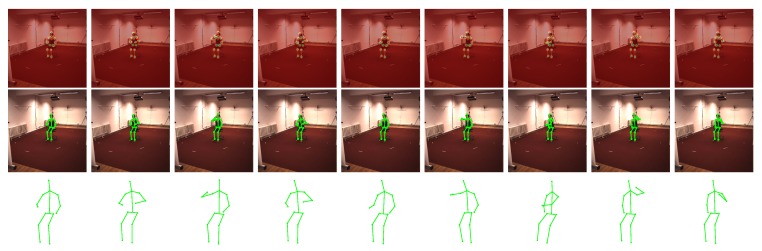
Evaluation of marker-less method on ’eating’ sequence in Human3.6M datasets. The visualization of results from nine selected frames are shown from left to right. For each frame, the heightmaps of all the joints are displayed together in the first row, the projection of our reconstructed 3D human structure is plotted in the second row and the reconstructed 3D structure is shown in the bottom row.

As described above, our method can be easily converted to the marker-less version. Cooperating with the CNN-based human joint detector, our method obtains accurate results as shown in Figure 6 and Figure 7. Note that the employed detector is not restricted to the one proposed in [32]. Any detector that provides the heightmaps can be introduced into our framework. This proves that our method is effective in more practical scenarios.

### 5.4. Discussion

Our method is also limited by the reconstructability problem, the same as the previous NRSfM techniques. When the input sequence has a small range of viewpoints and the human body is extremely small, the method yields poor reconstructions. In such cases, more additional priors are needed to obtain the robust 3D reconstructions, such as pre-learned shape basis, trajectory basis or temporal consistency. Moreover, camera rotation estimation is not taken into account in this paper. Similar to the method in [31], how to optimize the camera rotations along with estimating the 3D structures under the assumption, i.e., union of subspaces, is still an open problem.

## 6. Conclusions

In this paper, an NRSfM approach is proposed to recover complex 3D human motions from monocular RGB images. Kernelized low-rank representation, which empirically proves to be more effective to represent complex human motions, is introduced to the NRSfM framework. Combining with the soft inextensibility constraint, our method produces more accurate reconstruction against the baseline approaches. The qualitative analysis illustrates that our KLRR-NRSfM method can be conveniently converted to the mark-less version without dependency on the given tracks. However, some issues are still open for future research. For example, promoting the reconstruction accuracy is a challenging task when the range of viewpoints is extremely small, and estimating the camera rotations under the union of nonlinear shape subspaces assumption is also a future work, which is crucial for applying the method to more practical real-life scenarios. Additionally, the 2D locations of joints might be lost due to the occlusion. In the marker-based method, the 2D location of joints is often obtained by tracking the infrared markers. By contrast, in the marker-less case, the CNN-based detector is relatively robust. Nonetheless, an explicit mechanism is still needed to handle the occlusion for future works.

## Figures and Tables

**Table 1:** Quantitative evaluation on CMU motion capture sequences. The table reports the mean error $e_{mean}$ and median error $e_{med}$ of 3D reconstructions for the following NRSfM baselines: PTA [5], CSF [6], BMM [8], TUS [31], APG [9], CNR [14]; and our proposed method TUNS. For each sequence, the best and the second-best results of, both mean and median errors, are shown in red and blue, respectively.

	Method	PTA [5]	CSF [6]	BMM [8]	TUS [31]	APG [9]	CNR [14]	TUNS (Ours)
Data		$e_{\mathrm{mean}}/e_{\mathrm{med}}$	$e_{\mathrm{mean}}/e_{\mathrm{med}}$	$e_{\mathrm{mean}}/e_{\mathrm{med}}$	$e_{\mathrm{mean}}/e_{\mathrm{med}}$	$e_{\mathrm{mean}}/e_{\mathrm{med}}$	$e_{\mathrm{mean}}/e_{\mathrm{med}}$	$e_{\mathrm{mean}}/e_{\mathrm{med}}$
56_02		0.0227/0.0113	0.0500/0.0252	0.0235/0.0147	0.0204/0.0112	0.0215/0.0102	0.0341/0.0240	0.0205/0.0118
56_03		0.0655/0.0301	0.1309/0.0792	0.0748/0.0446	0.0557/0.0254	0.0739/0.0460	0.0605/0.0316	0.0448/0.0219
56_04		0.0720/0.0325	0.1819/0.1199	0.0843/0.0516	0.0637/0.0287	0.0792/0.0487	0.0661/0.0329	0.0538/0.0278
56_05		0.0697/0.0354	0.2056/0.1269	0.0857/0.0596	0.0613/0.0312	0.0741/0.0479	0.0629/0.0341	0.0506/0.0287
56_06		0.0951/0.0483	0.2412/0.1616	0.1085/0.0696	0.0827/0.0402	0.0975/0.0582	0.0829/0.0413	0.0667/0.0321
56_07		0.1259/0.0542	0.3446/0.2144	0.1453/0.0889	0.0959/0.0424	0.1262/0.0789	0.2066/0.0982	0.0790/0.0385
56_08		0.0807/0.0386	0.2015/0.1236	0.1158/0.0822	0.0717/0.0427	0.1415/0.1160	0.1910/0.1041	0.0583/0.0303
86_01		0.0700/0.0321	0.1591/0.0942	0.0832/0.0516	0.0619/0.0267	0.0642/0.0271	0.0607/0.0278	0.0582/0.0237
86_02		0.1817/0.0930	0.3170/0.2125	0.1716/0.0927	0.1521/0.0710	0.1586/0.0750	0.1463/0.0683	0.1449/0.0646
86_03		0.1861/0.1057	0.3696/0.2751	0.1766/0.1018	0.1554/0.0799	0.1578/0.0832	0.1541/0.0795	0.1433/0.0733
86_04		0.0934/0.0464	0.2384/0.1601	0.0975/0.0559	0.0826/0.0391	0.0854/0.0402	0.0783/0.0363	0.0774/0.0351
86_05		0.2267/0.1394	0.3387/0.2595	0.1975/0.1131	0.1821/0.0972	0.1872/0.1008	0.1738/0.0938	0.1784/0.0949
86_06		0.1765/0.0949	0.3724/0.2780	0.1745/0.0995	0.1570/0.0781	0.1617/0.0815	0.1516/0.0761	0.1501/0.0734
86_07		0.1457/0.0854	0.4838/0.3634	0.1447/0.0878	0.1293/0.0702	0.1331/0.0724	0.1254/0.0677	0.1253/0.0688
86_08		0.1333/0.0746	0.3672/0.2562	0.1366/0.0836	0.1191/0.0631	0.1227/0.0657	0.1152/0.0608	0.1157/0.0609
86_09		0.0302/0.0135	0.0728/0.0498	0.0412/0.0237	0.0306/0.0118	0.0310/0.0112	0.0291/0.0107	0.0270/0.0096
86_10		0.0636/0.0334	0.2489/0.1609	0.0681/0.0431	0.0534/0.0257	0.0552/0.0261	0.0516/0.0245	0.0505/0.0232
86_11		0.0727/0.0416	0.4362/0.3212	0.0729/0.0465	0.0605/0.0319	0.0630/0.0329	0.0586/0.0306	0.0569/0.0304
86_12		0.1190/0.0667	0.2714/0.2062	0.1225/0.0735	0.1102/0.0596	0.1131/0.0616	0.1096/0.0597	0.1078/0.0584
86_13		0.0676/0.0420	0.1261/0.0931	0.0777/0.0566	0.0586/0.0361	0.0684/0.0462	0.0584/0.0348	0.0515/0.0276
Average Err.		0.1049/0.0560	0.2579/0.1790	0.1101/0.0670	0.0902/0.0456	0.1008/0.0565	0.1008/0.0518	0.0830/0.0417
Relative Err.		1.2639/1.3429	3.1072/4.2926	1.3265/1.6067	1.0867/1.0935	1.2145/1.3549	1.2145/1.2422	1/1

**Table 2:** Quantitative evaluation on UMPM sequences. The table reports the mean error $e_{mean}$ and median error $e_{med}$ of 3D reconstructions for the following NRSfM baselines PTA [5], CSF [6], BMM [8], TUS [31], APG [9], CNR [14]; and our proposed method TUNS. For each sequence, the best and the second-best results of, both mean and median errors, are shown in red and blue, respectively.

	Method	PTA [5]	CSF [6]	BMM [8]	TUS [31]	APG [9]	CNR [14]	TUNS (Ours)
Data		$e_{\mathrm{mean}}/e_{\mathrm{med}}$	$e_{\mathrm{mean}}/e_{\mathrm{med}}$	$e_{\mathrm{mean}}/e_{\mathrm{med}}$	$e_{\mathrm{mean}}/e_{\mathrm{med}}$	$e_{\mathrm{mean}}/e_{\mathrm{med}}$	$e_{\mathrm{mean}}/e_{\mathrm{med}}$	$e_{\mathrm{mean}}/e_{\mathrm{med}}$
p1_grab_3		0.1036/0.0639	0.1619/0.1127	0.0976/0.0673	0.0882/0.0539	0.0908/0.0558	0.0805/0.0492	0.0781/0.0560
p1_chair_2		0.0763/0.0461	0.1889/0.1281	0.0892/0.0664	0.0736/0.0429	0.0736/0.0433	0.0678/0.0404	0.0628/0.0441
p3_ball_12		0.0431/0.0244	0.0930/0.0682	0.0709/0.0591	0.0423/0.0245	0.0414/0.0225	0.0407/0.0226	0.0286/0.0168
